# Societal output and use of research performed by health research groups

**DOI:** 10.1186/1478-4505-8-30

**Published:** 2010-10-12

**Authors:** Sebastian P Mostert, Stéfan PH Ellenbroek, Ingeborg Meijer, Gerrit van Ark, Eduard C Klasen

**Affiliations:** 1 Technopolis Group the Netherlands, Herengracht 141, 1015 BH Amsterdam, the Netherlands; 2 Directorate of Research, Leiden University Medical Center (LUMC), P.O. Box 9600, 2300 RC Leiden, the Netherlands; 3 Strategic Policy and Board Affairs, The Netherlands Organisation for Health Research and Development (ZonMw), P.O. Box 93 245, 2509 AE The Hague, the Netherlands; 4 Executive Board, Leiden University Medical Center (LUMC), P.O. Box 9600, 2300 RC Leiden, the Netherlands

## Abstract

The last decade has seen the evaluation of health research pay more and more attention to societal use and benefits of research in addition to scientific quality, both in qualitative and quantitative ways. This paper elaborates primarily on a quantitative approach to assess societal output and use of research performed by health research groups (societal quality of research). For this reason, one of the Dutch university medical centres (i.e. the Leiden University Medical Center (LUMC)) was chosen as the subject of a pilot study, because of its mission to integrate top patient care with medical, biomedical and healthcare research and education. All research departments were used as units of evaluation within this university medical centre.

The method consisted of a four-step process to reach a societal quality score per department, based on its (research) outreach to relevant societal stakeholders (the general public, healthcare professionals and the private sector). For each of these three types of stakeholders, indicators within four modes of communication were defined (knowledge production, knowledge exchange, knowledge use and earning capacity). These indicators were measured by a bottom-up approach in a qualitative way (i.e. all departments of the LUMC were asked to list all activities they would consider to be of societal relevance), after which they were converted into quantitative scores. These quantitative scores could then be compared to standardised scientific quality scores that are based on scientific publications and citations of peer-reviewed articles.

Based on the LUMC pilot study, only a weak correlation was found between societal and scientific quality. This suggests that societal quality needs additional activities to be performed by health research groups and is not simply the consequence of high scientific quality. Therefore we conclude that scientific and societal evaluation should be considered to be synergistic in terms of learning for the future, accountability and advocacy.

This quantitative approach to assess societal quality in a quantitative sense is based on indicators that function as proxies for society quality on different levels, based on the communication of researchers with their societal stakeholders (i.e. knowledge production, knowledge exchange and knowledge use). The methodology presented is just a first attempt to compare scientific quality scores (publication and citation scores) with societal quality scores in a quantitative way. This comparison can be used by organisations (e.g. university medical centres) in their planning and control cycle.

## Introduction

Research evaluation is meant to learn from, to account for and to advocate research performance [1-3]. Research evaluation developed in the last fifty years from measuring resources (inputs), policy and management (throughputs) and products (outputs) [4]. Also scientific use (scientific references) [3,5] and benefits of research results in the research community were included [6,7]. It is from the 1990s that societal products (outputs) [8-10], societal use (societal references) and societal benefits (changes in society) also came into scope [11-21].

This study was set up within a Dutch university medical centre: the Leiden University Medical Center (LUMC). University medical centres in the Netherlands are the result of mergers between university hospitals and medical faculties of universities, and combine university patient care with medical, biomedical and healthcare research and the education and training of doctors, medical researchers and other healthcare workers. The study was limited to research group evaluation. A research group is the minimal unit responsible for the scientific process as a whole: its resources, its direction and focus (policy), its organisation (management), its productivity and its impact on science and society. The study further focuses on societal outputs and use (societal quality) as an addition to scientific outputs and use (scientific quality). The study concentrates on transversal evaluation of research group performance rather than longitudinal evaluation of the research itself. Real societal impact (i.e. the increased state of public health) was not included in this study because it is virtually impossible to attribute public health changes to just one research group in the relatively short time frame of a transversal evaluation [22].

Research aims at the production of new knowledge and new skills that can be used in society [23-25]. Therefore, new knowledge is taught to students, is written down in scientific publications (formal knowledge) and professionals are trained with new skills (tacit knowledge). Research has both short-term (applied research) as well as long-term practical objectives (basic research). In general, basic research is considered to be an important source of new ideas (leads) for applied research, which in turn creates the societal basis for basic research [26-28]. This notion is effectively exploited by medical charities [29,30]. In health research this might be obvious, however until recently it has not been translated into evaluation practice [31]. Medical faculties devoted to basic research were evaluated on the basis of the scientific value of their research and the societal value of their teaching. Academic hospitals were evaluated on their innovative health care performance and their medical speciality training.

This situation has changed dramatically in the Netherlands over the last decade once all eight medical faculties merged with their local academic hospitals into eight University Medical Centers (UMCs). In addition, the medical research department of the Dutch Research Council (NWO-MW), which was mainly devoted to basic medical research, merged with the research organisation (ZON) of the Ministry of Health, Welfare and Sport, which was more focussed on health care research and innovation. The resulting organisation, ZonMw (Netherlands Organisation for Health Research & Development) is now the health component of the science system as well as the R&D component of the public health (innovation) system. As such, science and innovation systems for medical and health research have now effectively become integrated in the Netherlands.

The ultimate objective of health research is to improve the health outcome of each individual [32,33]. According to the Council for Medical Sciences of the Royal Netherlands Academy of Arts and Sciences (KNAW), health research has a dual mission, namely a scientific and a societal one: *"it is explicitly concerned not only with the acquisition of scientific knowledge as such, but also with the usefulness and implementation of scientific achievements" *[14]. The interaction of health research with society (health care, health industry and the general public) needs to be intensified, in order to accelerate and enhance effectiveness of the health innovation system and to enhance public support for health research.

Despite the societal character of health research, the performance of (bio)medical researchers still tends to be measured by the scientific quality of their research rather than by its use by the health care sector and its benefits to society at large. The funding agencies of health research in the Netherlands, the UMCs, the research organisations and individual researchers all strive for high scientific and societal quality, but current systems for evaluating the performance of research still focus on scientific quality. In 2001, the KNAW stated that *"a new evaluation instrument is needed for measuring societal quality" *[14]. Both quantitative and qualitative approaches to research evaluation can be used to connect the aims of health research to its perceived value of research outcomes, whether this is tied to scientific excellence or usefulness. In view of the tension between quantitative metrics being seemingly objective and external peer review being possibly subjective, a middle ground where quantitative quality measures are supported by peer review and vice versa should be sought for so that the strengths of each approach may compensate for the limitations of the other [19,34,35].

In recent years, a limited number of methods, indicators and criteria to assess the societal quality of fundamental, strategic and applied research have been developed. One method is the *Sci_Quest method *[36] that investigates the relationships between a research institute and its relevant context such as society, policy, and industry (stakeholders), which is applicable to all research domains. Another method is the *Payback framework *for assessing the impacts of health (care) research, which consists of a multidimensional categorisation of benefits and a model of how to assess them [37,30,38]. The *Payback framework *has been applied in the evaluation of the Health Care Efficiency Research programme of ZonMw [39]. In both 2007 and 2009, an international workshop was organised by the Swedish Research Council to discuss the concept of evaluation of socio-economic impacts from medical/health research. Recently an international and interdisciplinary consortium (SIAMPI) of knowledge institutions supported by the European Commission (within the Seventh Framework Programme) was created with the aim of developing methods for the assessment of social quality of research and research funding instruments. Given the context of the Dutch culture favouring outcome metrics, and the search for simplicity in research assessment, the methodology presented here builds on metrics first, while acknowledging that qualitative procedures need to be added in order to eventually reach an intelligent and advanced combined system of health research evaluation.

## Methodology

A first attempt to describe how to assess and compare scientific and societal quality of (health) research within one integrated framework was published by Van Ark and Klasen in 2007 [22]. In 2007, the framework was put into practice in a pilot study within the LUMC. The pilot study met the objective of the Executive Board to make the societal profile of the LUMC explicit in accordance to its mission statement.

The framework of Van Ark and Klasen is based on the concept of communication. In communication theory target groups and modes of communication are differentiated [7]. In that view, the outputs of a research groups depend on the quality and quantity of the communication (outreach) with relevant target sectors. Therefore, scientific quality essentially depends on the communication with the scientific community and societal quality depends on the communication with different societal sectors. Even though the interaction between science and society is increasing, it is often realised at the level of research programming, rather than at the level of research execution.

We define three types of societal sectors (or stakeholders): the general (lay) public, healthcare professionals and the private sector. In these sectors respectively cultural, social and economic outputs of research will appear. In health research, the *general public *is represented by the (mass) media. Healthcare professionals represent the healthcare system itself, including public health, health policy and health insurance systems. The *private sector *mainly consists of the human pharmaceutical and biotechnological, the medical ICT, medical devices and medical services sectors.

As modes of communication we use the same categories for both scientific and societal quality: knowledge production, knowledge exchange, knowledge use and earning capacity. *Knowledge production *is presented in a communicable form such as publications in all kind of media (scientific, professional or lay literature, radio, TV, papers, internet), services and/or products. Communication can also be in the form of direct *knowledge exchange *such as lectures and courses (from sender to receiver), conferences and external participation in research (group) guidance and governance (mutual interaction) and/or recognitions of repute (from receiver to sender) such as invited lectures and courses, awards, prizes and invited functions of researchers in external bodies. When knowledge products are transferred effectively to the stakeholders, *knowledge use *can arise as external citation of publications in the media mentioned, the external purchase and returns on products and services delivered. As an expression of the external value of a research group, the attraction of external resources or funds can be used: e.g. charity funding as a measure of perceived quality by the general public, research council and European funding as a measure of healthcare professional quality and private funding as a measure of quality for the private sector. Therefore the *magnitude *of the *earning capacity *shows to what extent the societal stakeholders are willing to contribute to the research [22]. While research income is often used as a proxy for research excellence, here we consider it as linking societal quality to scientific merit.

Benefits such as a contribution to the knowledge base in medicine (scientific), improvements in public health (social), prosperity (private) and public understanding (cultural) tend to take decades to develop and are virtually impossible to attribute to a single research group only [40,41]. Therefore, we have left scientific and societal impact of research (groups) out of the scope of this study as mentioned before, and discuss the level of societal outputs as a measure for societal quality only. However, we anticipate that in order to create societal *impact*, societal outputs are a *sine qua non*.

The pilot study followed a step-by-step procedure. It was set up so as to reach a good understanding of the concept of societal evaluation in addition to scientific evaluation in the near future, to explain the purpose of the pilot study to internal and external stakeholders and to aim for proper participation. The first stage consisted of a thorough inventory of two research groups with a good understanding of societal evaluation and willingness to participate in the pilot. These research groups were asked to deliver all kinds of information that they judged to be relevant. The data received was used to guide the second stage: an inventory of 43 departments, reflecting all research groups of the LUMC. An introductory letter from the Executive Board accompanied the voluntary inventory, to explain the scope and purpose of the pilot study and to ask for cooperation. The inventory was semi-structured consisting of two parts. In the predefined part, societal outreach data could be introduced in terms of indicators for societal knowledge production, knowledge exchange and knowledge use. In the open part, societal quality related data that the participants found to be relevant but which were not represented in the predefined part could be introduced and clarified. The response rate was 19/43 (45%) of the departments, representing approximately 60% of the LUMC's research population. For all 43 departments the existing data on scientific quality (produced for many years by the Centre for Science and Technology Studies (CWTS) and accepted by the departments) and earning capacity were added from the administrative database.

In the third and final analysis stage, all data was lumped into 23 indicator categories. Per indicator category, an adjustable weighting factor was introduced in order to differentiate between the importance of the chosen indicators. It was then possible to enter the 23 indicator categories into a 12-cell matrix consisting of 3 columns representing the target sectors and 4 rows representing the modes of communication (see Table 1).

**Table 1:** Societal quality indicator matrix

	General public	Healthcare professionals	Private sector
**Knowledge production**	• Contributions to television programmes • Contributions to radio programmes • Contributions to newspapers or journals (non peer reviewed) • Contributions to public websites • Contributions to publics news forums • Contributions to schoolbooks or study material	• Publications in medical journals (non peer reviewed) • Contributions to professional websites • Contributions to medical charters or protocols	• Patents

**Knowledge exchange**	• Memberships of public funding agencies or patient organisations • Speeches for general public or contributions to public forums • Information for scholars	• Memberships of advisory committees or professional associations • Speeches at medical conferences	• Speeches for companies • Cooperation with companies

**Knowledge use**	• Use of schoolbooks or study material in medical education programmes	• Use of new medical charters or protocols in medical practice for diagnosis or therapy	• Use of technology by companies to produce new products or therapies

**Earning capacity**	• Charity funding (3^rd ^money stream)	• Indirect funding (2^nd ^money stream)	• Contract funding (4^th ^money stream)

This table lists the indicators that are used in this pilot study for each of the three target groups and modes of communication

## Results

This section deals with a description of the procedure undertaken in order to acquire and quantify the societal outreach of the departments. In addition to this description, the validation of the methodology used and its utility for institutional policy are taken into consideration.

### Data acquisition and quantification

All reported societal outreach was categorised and counted per department. Here we describe the use of weighting factors to adjust the importance of the indicators that are used, and the way to quantify the obtained qualitative dataset. The calculation of the quantitative societal quality score consists of four discrete steps, which will be explained below. The 23 (bottom up) categories are considered to be suitable indicators (see Table 1).

#### Step 1 - Indicator occurrences

In order to be able to compare the results of the LUMC departments mutually, the number of occurrences of each indicator (e.g. the number of contributions to television programmes, the number of contributions to medical charters or protocols or the number of patents) for every single department is related to the average number of occurrences of that indicator for all the departments.

#### Step 2 - Weighting system

When the number of occurrences of the indicators is related to the average of all the departments, the next step is to calculate the weighting factor for each indicator. The advantage of our method is that the indicators can be weighted differently, dependent of the objectives of the research group. To include a weighting system, first a weighting score has to be calculated. For each sector (the general public, healthcare professionals and the private sector), an analysis is made of how indicator A ranks in relation to indicators B, C, D, and so on. In this way it is possible to calculate a relative weighting score for each indicator (within each sector), i.e. whether the indicator is more or less important than the other indicators.

The relative weighting score is the input for the calculation of the real weighting factor that indicates the influence of a certain indicator compared to the others. In addition to the option to give some indicators a higher weighting factor, it is also possible to include weighting factors between the three sectors to adjust for the importance of a particular sector. In this pilot study, the authors have defined the weighting factors. In future iterations the weighting factors can be defined by research groups themselves or the Executive Board.

#### Step 3 - Societal quality per indicator

After the creation of the weighting system, the actual calculation of the societal quality per indicator can be conducted. Per department and for each indicator this specific societal quality is the result of step 1 (the relative occurrences of each indicator) and step 2 (the weighting factor for each indicator). Societal quality per indicator is ideally directly related to the mission and objectives of a research group and can be adjusted accordingly. Mission, objectives and, if possible, indicators should relate to perceived benefit by end users.

#### Step 4 - Societal quality per target group and total societal quality

Together, the 23 individual societal quality indicators form the basis for the calculation of the societal quality for each sector and the total societal quality. The calculation of the general public, healthcare professionals and the private sector societal quality is essentially similar: for all indicators in that particular sector the average of the indicator societal qualities is taken. The total societal quality for the departments is obtained by taking the average of the three sectoral societal quality scores.

Because the weight of all indicators is related to the number of occurrences (the uniqueness = step 1) as well as a ranking of all indicators (the importance = step 2), where the arbitrary weighting between the different sectors only takes place in the last step (4), then the robustness of the system is greatly enhanced.

**Figure 1:**
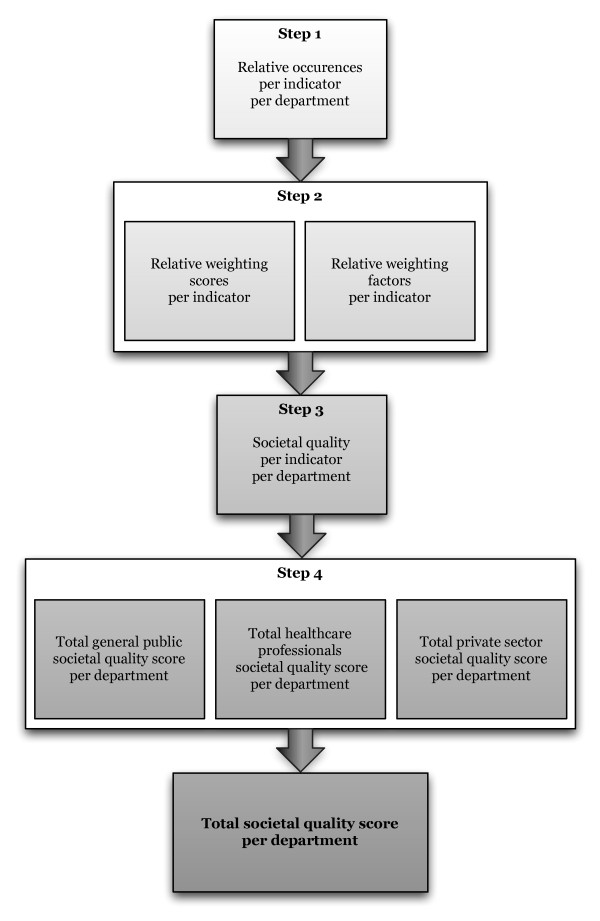
**Societal quality calculation steps**. This figure shows schematically the subsequent steps that are required to calculate the total societal quality score of each individual department.

The following figure (Figure 1) presents the different steps in the process of calculating the societal quality of a research group.

**Figure 2:**
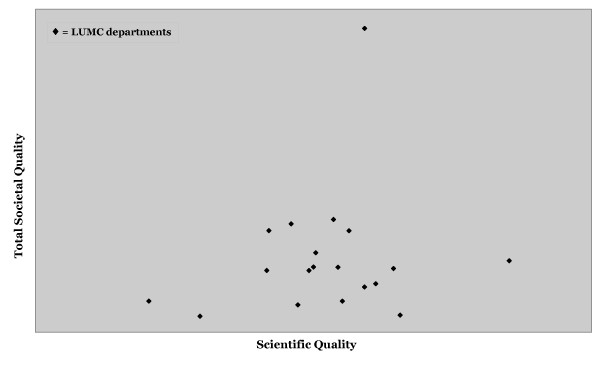
**Total Societal Quality versus Scientific Quality**. In this graph the Total Societal Quality is plotted against the Scientific Quality of all LUMC departments that contributed to this study. The correlation between the two is weak which suggests that societal quality is not simply the consequence of high scientific quality.

When comparing the total societal quality to the scientific quality scores of the departments, it is shown that correlation between the two is weak (see Figure 2). This suggests that high scientific quality of research groups is not necessarily related to communication with society, and that in order to increase societal quality of research groups, additional activities are needed. Therefore societal quality is not simply the consequence of high scientific quality. Obviously, in a university medical centre, scientific quality prevails, and is a prerequisite, which cannot be replaced by aiming instead for high societal quality.

**Figure 3:**
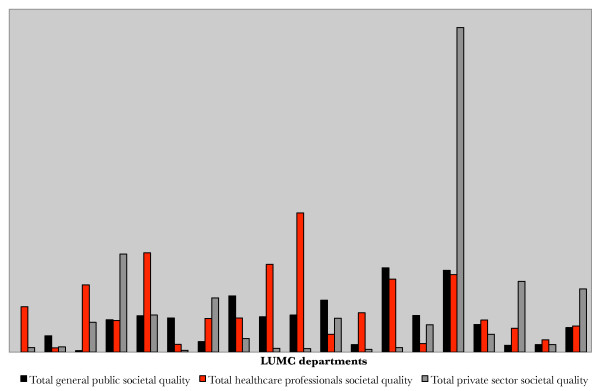
**Total target group societal quality scores per department**. The graph shows the societal quality scores per department for each of the three target groups (i.e. the general public, healthcare professionals and the private sector).

Because of the weighting of indicators, the method can be adjusted to the objectives of the research group. For example, when a research group's objective is more focused on communication with the private sector, the indicators for private sector societal impact can be valued as more important than the indicators for healthcare professionals societal quality. Therefore, through these adjustments, the effects of the quantitative measurements will reflect the objectives of the research group on their outreach (if present). The following figure (Figure 3) presents the results of the pilot study for each of the target groups. Based on this graph it can be concluded that some departments focus more on private rather than healthcare professionals or the general public and the other way around.

It should be noted here that in the quantification process, i.e. the calculation of societal quality, the size of a research group is not included. It is anticipated that this may influence the results, however it was left out because in a university medical centre, the people on the payroll of a department do not spend all of their time on research since most departments also have to perform education and care. In cases where it is not possible to properly define the number of research 'units', calculations would be misleading.

### Validation of the method

Given the lack of a gold standard, the first subjective validation of the methodology was to see whether the resulting sectoral societal qualities were in keeping with the annual assessments of the Executive Board (supported by the LUMC central science committee) and the latest (2006) external visitation on the departments involved. This was generally the case, although occasionally the score of departments deviated slightly. The method proves that a high total societal quality score can only be achieved if a department shows some activity in all sectors. Given that a strong push of the interests of industry and commerce should be counterbalanced by a pull toward broader public communication (and vice versa), this was deemed fair, as a high score for total societal quality should be for those departments that are aware of their potential role in all sectors of society. In addition, departments that are capable of attracting external money achieve high scores as well, which reflects that they are recognised in society as internationally leading groups in a relevant clinical field.

Secondly, the reliability of the method was tested by changing the values of the different weighting factors. Even rather major changes to the weighting factors did not really change the results of the test. This is due to the fact that a total of 23 separate indicators are correlated to one another. This shows that the exact choice of indicators and their relative weight is somewhat irrelevant as long as a stable quantitative scheme is chosen.

A more objective, external validation was performed in collaboration with the Netherlands Organisation for Health Research and Development (ZonMw). The LUMC receives grants from ZonMw on the basis of *ex-ante *assessment of their scientific quality and its relevance for the research programme. Within ZonMw different sub-departments fund different types of research. We could demonstrate that departments receiving grants primarily from the Science and Innovation sub-department had a higher score on commercial impact and that the departments primarily receiving grants from other sub-departments had a higher score on societal quality within the sectors general public and healthcare professionals. This reflects the priority differences that exist between those separate sub-departments of ZonMw. However, there is no difference in general societal quality of the LUMC departments that receive funding from the different ZonMw sub-departments.

## Conclusions

The communication metaphor has been shown to be useful for introducing societal quality in addition to scientific quality evaluation in an academic environment accustomed to and convinced of the sense and usefulness of the latter but not necessarily of the first. Essentially, in a university medical centre scientific quality prevails, and is considered to be a prerequisite for societal quality. Still, when comparing the total societal quality to the scientific quality scores of the LUMC departments, it is shown that correlation between the two is weak. This suggests that societal quality needs additional activities and is not simply the consequence of high scientific quality.

Our appraisal is therefore that evaluation of societal quality is as important as scientific evaluation: scientific and societal quality evaluation should be considered to be synergistic in terms of learning for the future, accountability and advocacy. The communication metaphor is useful to 'de-ideologise' the debate, showing that scientific quality and societal quality can be seen as two sides of the same coin. The communication metaphor has also been shown to be a good framework to start the rather new field of societal evaluation as it runs parallel to scientific evaluation.

We conclude from the pilot study that we have taken the first step on a long track to incorporate societal ex-post evaluation in addition to scientific ex-post evaluation of health research at the level of research groups. It has been shown to result in meaningful data that can be validated internally and externally. The results have been shown to be stable for small changes in the parameters of the counting scheme. It is necessary to continue these efforts to show the robustness of the method over time in the LUMC environment (validation in time), to show its usefulness in other university medical centre R&D environments in the Netherlands and abroad (contextual validation).

As a next step, it would be useful to include a normalisation of the size of a research group. In this pilot, large departments (more than 50 researchers) are directly compared to small departments (less than 20 researchers). It could be hypothesised that large groups have a greater capacity for outreach activities, which are more beneficial for societal quality than smaller departments. However, this should be investigated in more detail.

The methodology for societal ex-post evaluation at research group level is still at quite an early stage compared to the internationally accepted methodology for scientific evaluation of research groups as developed by CWTS that all Dutch UMCs adhere to. This method is based on normalised peer reviewed science citation impact analysis, which does not include earning capacity (or research income). Therefore, the order of magnitude of earning capacity is used as the only external valuation of the research conducted in the research group. This income however does not relate directly to any specific project because the quality is assessed at the level of the entire research group.

Follow-up research is needed to reduce and pre-define (and therefore remove the necessity of lumping bottom-up reported activities) the extensive set of indicators used in this pilot study to an internationally agreed, accepted and less laborious set, which would allow for automation of data collection. The stability of the calculation scheme suggests that the indicators tend to correlate with each other. This in principle opens the way to reduce the number of indicators.

However, the discussion on metrics and indicators typical for a quantitative approach, conceals the fact that this method does involve a qualitative phase as well. This includes feedback to the research groups, defining mission and objectives by research groups and simultaneous activities directed at one or more of the societal sectors, discussion on weighting factors, and peer review by external stakeholders in the case of an external evaluation. Therefore, we do not overestimate the value of quantifying and calculating with metrics, but instead seek to maintain a balanced approach. Even though 'metrics' have their place, and can make assessment of societal quality more efficient and cost-effective, their role is to signal, rather than be a direct replacement for qualitative assessments.

Because of the weighting of indicators, it is possible to adjust the method to the objectives of a research group. For example, when a research group's objective is more focused on outreach to the private sector, the indicators for private sector societal quality can be valued as more important than the indicators for societal quality with respect to healthcare professionals. The possibility to adjust the methodology to measure the effects reflecting the objectives of the research group is of added value. Ideally, a research group has a mission and objectives as to what they are aiming for with their research, both in scientific and in societal terms. But as long as they do not have explicit societal objectives, activities and outputs in this area are hard to adjust. Therefore, demonstrating the societal quality of medical research, even without a clear objective, stresses its value. This directly relates to the purpose of the evaluation, and the commissioning entity. Evaluations can be used for:

• justification (to produce data that can be used to justify or promote a programme);

• monitoring and measurement (count outputs, determine the efficiency and productivity of these outputs relative to the inputs); but also for

• learning (to understand what the programme is actually doing; how it is achieving/not-achieving its results); or

• management and improvement (to use learning from evaluation process in order to improve the programme).

In this case, the pilot study has been commissioned by the Executive Board of the LUMC, because they consider societal quality important on an organisational level. That being the case, reporting on this tends to be on an *ad-hoc *basis and usually focuses on success stories. The method described above is a means to get a better understanding of all interactions with society, i.e. serves a learning purpose. Additionally, the information from societal quality assessment is ultimately intended to be used in the regular planning and control cycle of organisations. This is deemed fair as for some disciplines, societal quality is at least as important as scientific quality or medical production.

However, the principal purpose of this pilot study is its contribution to creating awareness of what societal quality is, and how to make the concept operational, in a way that is not principally different and/or contradictory to evaluation of scientific quality.

## Discussion

The health science and innovation system in the Netherlands is currently at a unique stage of integration. All medical faculties are effectively integrated with their academic hospitals, which resulted in the creation of the University Medical Centers (UMCs). At the intermediate level, the research council (ZonMw) is the merger of the medical department of the Dutch Research Council (NWO-MW) and the innovation department of the public health policy system (the Ministry of Health, Welfare and Sport). Compared to the UK, ZonMw could be considered as the merger of the MRC and the R&D department of the NHS. It is against this context that the above study may be of value. It is an attempt to evaluate the societal quality of all health research, basic and applied. We are convinced that in modern medical and health sciences the distinction between basic (long-term) and applied (short-term) research will disappear. Increasingly basic research has short time practical consequences in the health care system and in privately owned health companies. Conversely, applied research and the implementation of established results often need a long-term approach. The fact that basic and applied health research systems do not act as an integrated system will be less acceptable to the general public. Also, more often the UMCs in the Netherlands consider the spin-off to the economic system as their societal obligation. Although this is encouraged by (Dutch) governmental departments, it is not yet realised at the intermediary level.

In comparison with other methods that are designed to measure societal quality, the methodology described here resembles the *Sci_Quest method *[36] in that the interaction with surrounding actors in the R&D system is taken as an important aspect. However, the *Sci_Quest method *has been mainly used at an institutional level or programme level, and includes other parts of the institutional mission (i.e. education and training) as part of the evaluation, whereas this model focuses at the research group level. Interaction and outreach is also measured in a different way: *Sci_Quest *reflects on the outputs by peer review with stakeholders and takes into account their perception whereas the model described here is limited to the activities and outputs of the research group itself. Like the *Sci_Quest method*, our evaluation methodology takes a transversal approach. This means that outputs measured are not necessarily related to the same piece or programme of research. This is different from the *Payback framework *[29,30], which takes a longitudinal approach in time that relates outputs and impacts to the same piece or programme of research, but not necessarily to the same actors. The *Sci_Quest method *and communication metaphor therefore eventually relate to the impact of research performers and the *Payback framework *to the impact of knowledge generation. As such, the three models complement rather than contradict each other and can be used for different levels of aggregation, time horizons and perspectives of research evaluation.

## Competing interests

The authors declare that they have no competing interests.

## Authors' contributions

SPM conducted the quantitative pilot study (i.e. the development of the indicators used and the calculation of societal quality) within the Leiden University Medical Center and drafted the manuscript. SPHE was responsible for the preparation of the data based on qualitative responses of the individual LUMC departments regarding their societal outreach, assisted in the quantitative pilot study and helped to draft the manuscript. IM participated in the methodological design and coordination of the study and helped to draft the manuscript. GA conceived the study and was responsible for the contextual background. ECK supported the study on behalf of the Executive Board of the Leiden University Medical Center. All authors read and approved the final manuscript.
